# First In Vivo ^23^Na Human Imaging at 10.5 T Using a Combined Sodium‐Proton Transceiver Body Array

**DOI:** 10.1002/mrm.70201

**Published:** 2025-12-02

**Authors:** Simon Schmidt, Arcan M. Ertürk, Gregory J. Metzger

**Affiliations:** ^1^ Center for Magnetic Resonance Research University of Minnesota Minneapolis Minnesota USA; ^2^ Medical Physics in Radiology German Cancer Research Center (DKFZ) Heidelberg Germany

**Keywords:** 10.5 T, body MRI, self‐gating, sodium MRI, ultra‐high field MRI

## Abstract

**Purpose:**

To demonstrate the first in vivo human ^23^Na MRI at 10.5 T using a novel dual‐tuned transceiver body array and to evaluate a self‐gating approach for respiratory motion compensation, focusing on renal imaging.

**Methods:**

A custom‐built eight‐channel ^23^Na‐loop ^1^H‐dipole transceiver array was designed, constructed, and characterized. Safe operation was ensured through comparison of electromagnetic simulations against B_1_^+^ and SAR phantom measurements. A programmable motion phantom mimicked respiratory motion to assess self‐gating for both the ^23^Na and ^1^H acquisitions. Finally, in vivo human abdominal ^23^Na and ^1^H data were acquired in three healthy volunteers under free breathing, applying the self‐gating approach.

**Results:**

The array's electromagnetic model showed good agreement with experimental data, with B_1_^+^ NRMSE values of 0.16 for ^23^Na, 0.32 for ^1^H, and SAR NRMSE values of 0.35 for ^23^Na and 0.22 for ^1^H. Total power limits of 125 and 42 W were implemented for ^23^Na and ^1^H, respectively. The motion phantom study confirmed that both ^23^Na and ^1^H self‐gating signals accurately tracked the programmed ground truth motion (correlation coefficients: 0.979 for ^23^Na, 0.995 for ^1^H). Motion binning significantly improved image sharpness as quantitatively evaluated in the phantom. In vivo, self‐gating robustly captured respiratory motion and effectively enabled motion compensation, as shown in binned images with enhanced anatomical detail.

**Conclusions:**

We successfully performed the first in vivo human ^23^Na MRI at 10.5 T. The developed dual‐tuned array and validated self‐gating technique enable 10.5 T imaging in humans, paving the way for quantitative studies of sodium homeostasis and advancing diagnostic capabilities.

## 1 Introduction

In general, sodium (^23^Na) MRI offers a unique perspective on the physiology and pathophysiology of various tissues and organs, complementing the structural information provided by conventional proton (^1^H) MRI. Unlike protons, sodium's role as a ubiquitous intracellular ion makes it a direct marker of cellular function, as it plays critical roles in numerous biological processes, including nerve impulse transmission, muscle contraction, and cellular homeostasis. By quantifying sodium concentrations and distributions, ^23^Na MRI can provide valuable diagnostic information for a wide range of diseases, including stroke [1, 2], brain tumors [3, 4], multiple sclerosis [5, 6], and muscle disorders [7, 8].

The kidneys play a central role in maintaining sodium homeostasis, which is crucial for regulating blood volume, blood pressure, and overall fluid balance in the body. Central to the organ's ability to concentrate urine and regulate fluid balance is the distinct sodium distribution in the kidneys, which is characterized by the corticomedullary gradient. This gradient increases linearly from the cortex to the medulla and is maintained by the selective reabsorption of sodium along the nephron. This gradient is dynamic and can be altered by physiological or pathological states, such as chronic kidney disease, acute kidney injury, and cardiorenal syndrome. It often presents as a reduced or flattened gradient compared to healthy individuals, which can be measured using ^23^Na MRI [9, 10, 11, 12].

Despite its immense potential, ^23^Na MRI faces significant technical challenges. Its low gyromagnetic ratio ($\gamma_{23\mathrm{Na}}$ = 11.26 MHz/T) and relatively low physiological concentrations (15–350 mM) result in a substantially lower signal‐to‐noise ratio (SNR) compared to ^1^H MRI. To overcome these limitations, various strategies have been explored, including the development of ultrahigh‐field (UHF) MRI systems, which offer improved SNR due to their increased magnetic field strength. The 10.5 T whole‐body MRI system at the University of Minnesota's Center for Magnetic Resonance Research represents a significant advancement in UHF MRI technology and offers a transformative opportunity for ^23^Na MRI, potentially unlocking new research capabilities. In this context of pushing magnetic field limits other pioneering UHF systems being developed and commissioned around the world [13, 14, 15] also represent critical, ongoing efforts to explore the capabilities of whole‐body UHF imaging. However, realizing this potential of UHF imaging above 10 T requires addressing several critical technical hurdles.

One of the most significant challenges is the design and implementation of efficient and safe radiofrequency (RF) coils. At 10.5 T, the shorter RF wavelengths and increased RF power deposition exacerbate the complexities of coil design [16, 17, 18, 19, 20]. Developing specialized RF coils capable of transmitting and receiving both ^23^Na and ^1^H signals with high efficiency and minimal specific absorption rate (SAR) is paramount. This necessitates optimized coil geometries, advanced electromagnetic simulations, and meticulous optimizations during the manufacturing process. Furthermore, the increased RF power deposition at UHF necessitates strict SAR management to ensure patient safety.

Motion artifacts, particularly those arising from respiratory motion, pose an additional substantial challenge in abdominal ^23^Na MRI. To mitigate these artifacts, self‐gating techniques are a promising tool [21, 22, 23, 24]. Platt et al.'s successful demonstration of a ^23^Na self‐gating approach at 7 T highlights the potential of this strategy for reducing image blurring [25]. Its translation to other field strengths and/or different coil designs, however, remains to be demonstrated, as well as a controlled investigation into its accuracy in capturing the respiratory motion with a known ground‐truth.

This manuscript reports the first successful in vivo human ^23^Na MRI at 10.5 T, achieved using a novel ^23^Na‐loop ^1^H‐dipole transceiver array. The array design enables acquisition of ^23^Na and ^1^H images within a single scanning session, facilitating precise anatomical co‐registration. We detail the design, construction, and characterization of the RF array, including comprehensive electromagnetic simulations and experimental measurements. In addition, we present phantom measurements using a motion phantom to evaluate the effectiveness of ^23^Na self‐gating techniques. Finally, we showcase in vivo results obtained from three healthy volunteers, specifically targeting the kidneys, demonstrating the feasibility and potential of human ^23^Na MRI at 10.5 T.

## 2 Methods

All MR experiments in this study were performed on a 10.5 T whole‐body magnet (Agilent Technologies, Oxford, UK) equipped with a whole‐body gradient coil (Siemens Healthineers, Erlangen, Germany).

### 2.1 Coil Array Design

**FIGURE 1:**
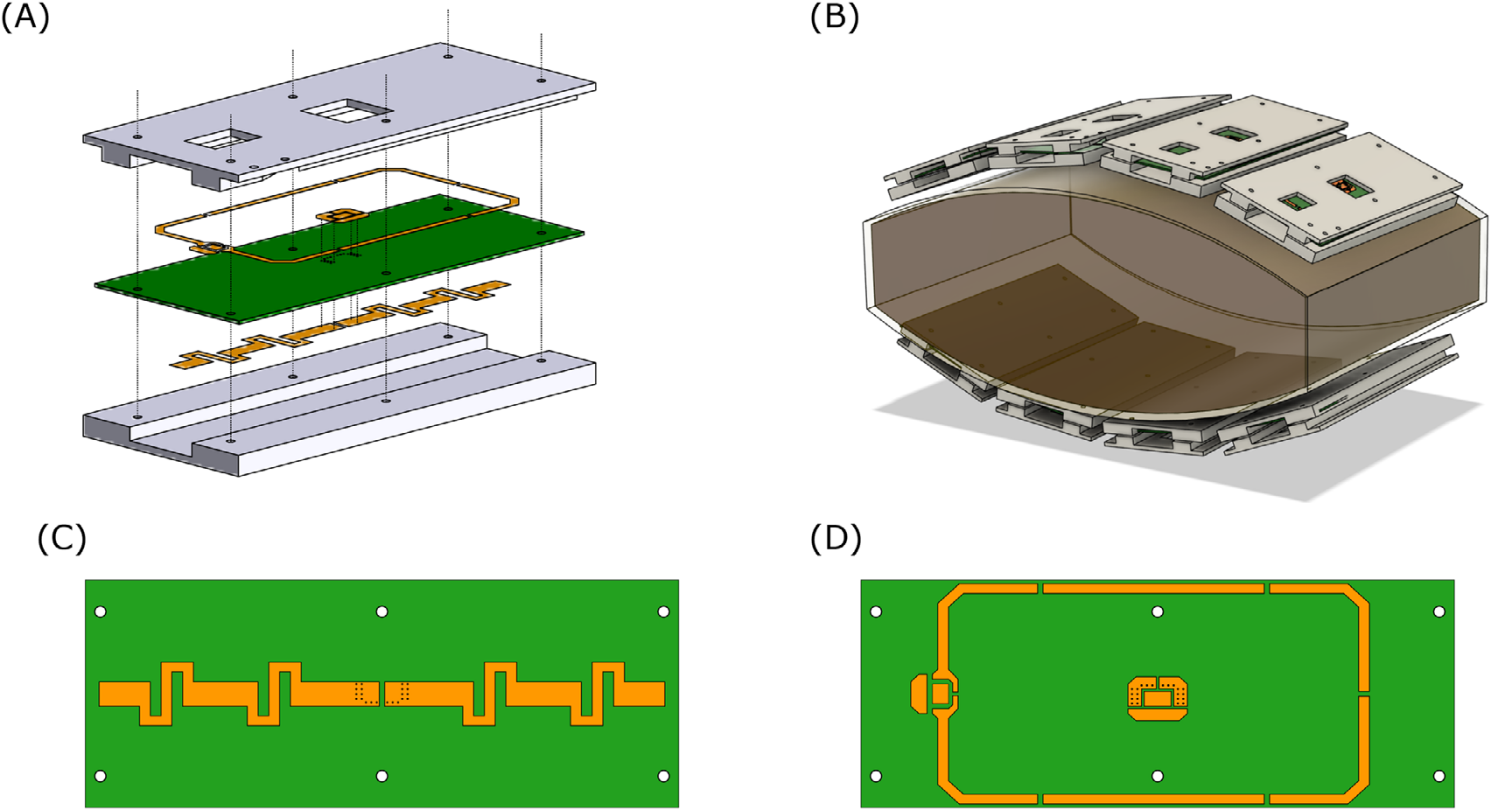
Overview of the ^23^Na‐loop ^1^H‐dipole transceiver body array. (A) Exploded view of a single array element, highlighting the double‐sided printed circuit board integrated within two 10‐mm‐thick housing blocks. (B) EM simulation model demonstrating the array's arrangement around a body‐sized phantom, as used for the safety assessment. (C, D) Detailed views of the printed circuit board's two sides: (C) the fractionated dipole for ^1^H and (D) the loop structure for ^23^Na, as well as the copper patches for the feeding network of the dipole.

The coil array design in this work is the result of previous work by Erturk et al. [26], presented at the 27th Annual Meeting of the ISMRM in Montreal, Canada, in 2019. The custom‐built coil array comprised eight identical modular blocks (Figure 1). Each block integrated a 20 cm long fractionated dipole antenna, resonant at the ^1^H Larmor frequency (447 MHz), and a 9 cm × 16 cm rectangular loop antenna, resonant at the ^23^Na Larmor frequency (118 MHz). The loops were aligned with the dipoles along their longitudinal axes to minimize coupling. Conductive traces of the loops and dipoles were implemented on double‐sided printed circuit boards, with dipoles situated on the load‐facing side and mounted between two 10‐mm‐thick blocks of thermoplastic polyetherimide (ULTEM 1000 resin, Sabic Global, Pittsfield, USA). The array was configured as an anterior and a posterior half, each containing four blocks housed within a flexible vinyl fabric with a center‐to‐center spacing of 12 cm. Due to limitations of the MRI system's architecture, separate eight‐way power splitters, tuned to the respective ^1^H and ^23^Na resonance frequencies, were employed for signal transmission to the array elements. Both splitters introduced a phase increment between adjacent output channels of approximately 45°, enabling the excitation of different circularly polarized modes (CP^+^, CP^2+^, …) depending on the element connection order.

### 2.2 Coil Safety Evaluation

The electromagnetic (EM) safety of the coil array was evaluated using a Tier 3 validation approach [27], consistent with our previously published work on a 10.5 T 16‐channel ^1^H body coil [18]. This involved experimental characterization of B_1_^+^ fields and SAR for both ^1^H and ^23^Na in a polyvinylpyrrolidone (PVP)‐agar phantom (651 g PVP, 15 g agar, 17.8 g NaCl, and 0.47 g NiCl_2_ per 1000 g H_2_O) with a torso‐approximating cross‐section of 190 mm (height) by 450 mm (width) (The Phantom Laboratory, Salem, USA).

Absolute B_1_^+^ mapping for ^23^Na was performed using a series of gradient‐echo images with varying reference voltages of 100–500 V in steps of 50 V (while keeping the nominal flip angle fixed at 90°) and subsequent fitting of a sinusoidal signal model. For ^1^H, the AFI method was employed, targeting a flip angle of 45°. Experimental SAR distributions were determined via MR thermometry using multi‐echo gradient‐echo data (TE = 5, 10, and 15 ms) acquired at the ^1^H frequency before and after a 10‐min heating sequence. Heating was induced by transmitting on either the ^1^H or ^23^Na elements with average powers of 119 and 145 W, respectively. Thermometry data were corrected for a spatially uniform offset using a small oil‐filled reference within the phantom. SAR was calculated from the measured temperature increase (∆T) as $\mathrm{SAR}=c_{p}\cdot ∆T/∆t$, where $c_{p}=3500\;J/\mathrm{kg}/K$ is the specific heat capacity of the phantom material (measured with KD2 Pro Thermal Properties Analyzer, Decagon Devices Inc., Pullman, USA) and ∆t=600s is the heating duration. Finally, SAR values were normalized by the incident time‐averaged power.

A virtual model of the coil array was generated in Sim4Life (Zurich Medtech, Zürich, Switzerland) based on a CT scan of the experimental setup. Channel‐wise E‐ and B‐fields were computed using the integrated Finite‐Difference Time‐Domain (FDTD) solver. Following channel‐wise real‐valued scaling of the simulation results to match experimentally measured channel‐wise B_1_^+^ magnitudes, the simulated and experimental B_1_^+^ and SAR distributions were compared.

To establish safe operating power limits, the virtual coil array was positioned over the kidneys in three distinct anatomical human body models [28, 29] (Duke, Ella, and Fats29). The peak 10 g SAR was evaluated for both ^1^H and ^23^Na using the determined relative phases from the eight‐way splitters across all models. Total power limits were subsequently determined based on the maximum peak 10 g SAR values under consideration of the maximum permissible SAR value of 20 W/Kg in the first level control mode.

### 2.3 Motion Phantom

**FIGURE 2:**
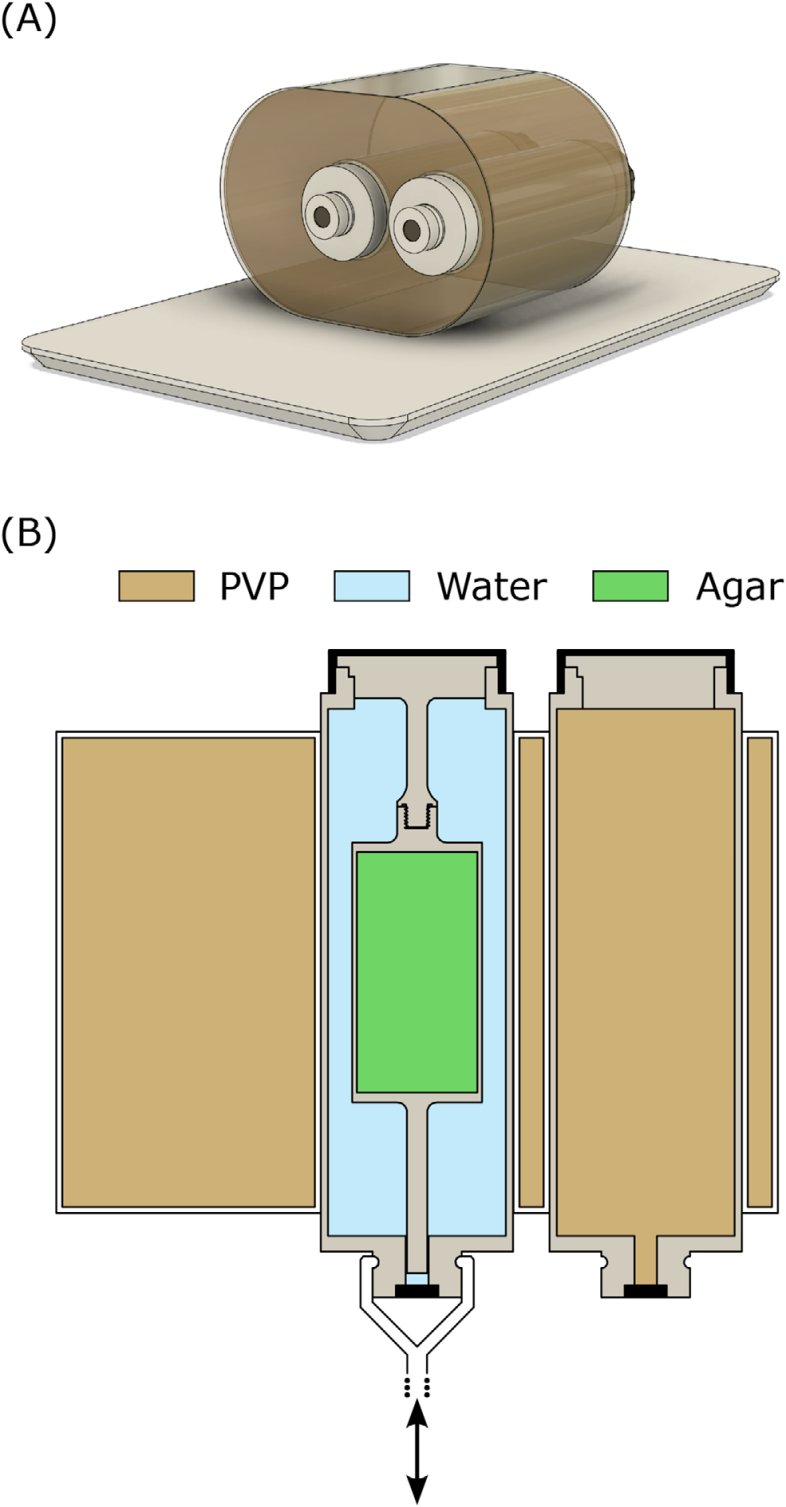
Overview and internal structure of the motion phantom. (A) 3D rendering of the motion phantom, illustrating its modular design consisting of a static oval housing and two cylindrical inserts. (B) Coronal cross‐section schematic of the assembled phantom. This view details the internal arrangement, featuring a 3D‐printed simplified kidney model represented by an agar‐filled cylinder (green) concentrically positioned within a water‐filled (light blue) chamber. The bottom section of the phantom indicates the coupling point for external motor actuation, with an arrow signifying the direction of motion.

To evaluate the self‐navigation capabilities of the new coil array under controlled motion, we utilized an MR‐compatible programmable motion phantom (QUASAR MRI 4D Motion Phantom, Modus Medical Devices, London, Canada) featuring a stationary oval body and two cylindrical inserts (as depicted in Figure 2). One insert and the stationary body were filled with a tissue‐mimicking solution of 537 g PVP, 9.3 g NaCl, and 0.47 g NiCl_2_ per 1000 g H_2_O. The second insert, driven by an MR‐compatible motor assembly, contained a custom‐designed 3D‐printed part. This part consisted of a cylinder filled with a 4% agar solution (175 mM sodium concentration) to mimic a simplified kidney model, surrounded by deionized water. To simulate realistic physiological motion, a 30‐min respiratory signal, previously recorded in vivo using a respiratory belt, was loaded into the motion phantom's control software to drive the moveable insert.

Data acquisition was performed with the ^23^Na–^1^H array tightly positioned around the motion phantom. Two separate acquisitions were performed for both nuclei using a 3D FLASH sequence with a center‐out radial koosh‐ball trajectory with a 2D golden means pattern [30]: one under static conditions with the cylindrical insert centered within the static phantom body, and a second with the insert undergoing motion driven by the prerecorded respiratory waveform. To enable retrospective self‐gating, additional ADC samples were acquired at the k‐space center before the readout gradients. Detailed acquisition parameters are summarized in Table 1.

**TABLE 1:** Acquisition parameters.

	^23^Na motion phantom study	^1^H motion phantom study	^23^Na in vivo study	^1^H in vivo study
TR (ms)	10	10	10	10
TE (ms)	0.98	0.46	0.94	0.47
Nominal FA (°)	36	10	32	17
FOV (mm^3^)	320 × 320 × 320	320 × 320 × 320	500 × 500 × 500	500 × 500 × 500
Resolution (mm^3^)	3.0 × 3.0 × 3.0	1.5 × 1.5 × 1.5	4.5 × 4.5 × 4.5	2.25 × 2.25 × 2.25
BW (Hz/Px)	333	854	250	659
Projections	185 000/60 000	185 000/60 000	180 000	180 000
Self‐gating ADC points	10	10	6	10
TA (min)	30:50/10:00	30:50/10:00	30:00	30:00

Abbreviations: BW, (readout) bandwidth; FA, flip angle; TA, time of acquisition.

Data reconstruction was performed offline using MATLAB (R2021a, The Mathworks Inc., Natick, Massachusetts, USA) and the Berkeley Advanced Reconstruction Toolbox [31] (BART). The reconstruction pipeline comprised the following steps:

1. Density compensation of the acquired k‐space data.
2. Binning of k‐space data into distinct bins based on the self‐gating signal.
3. Nonuniform Fast Fourier Transformation (NUFFT) using BART.
4. Receive sensitivity profile estimation (ESPIRiT [32]) using BART.
5. SENSE1 reconstruction

**FIGURE 3:**
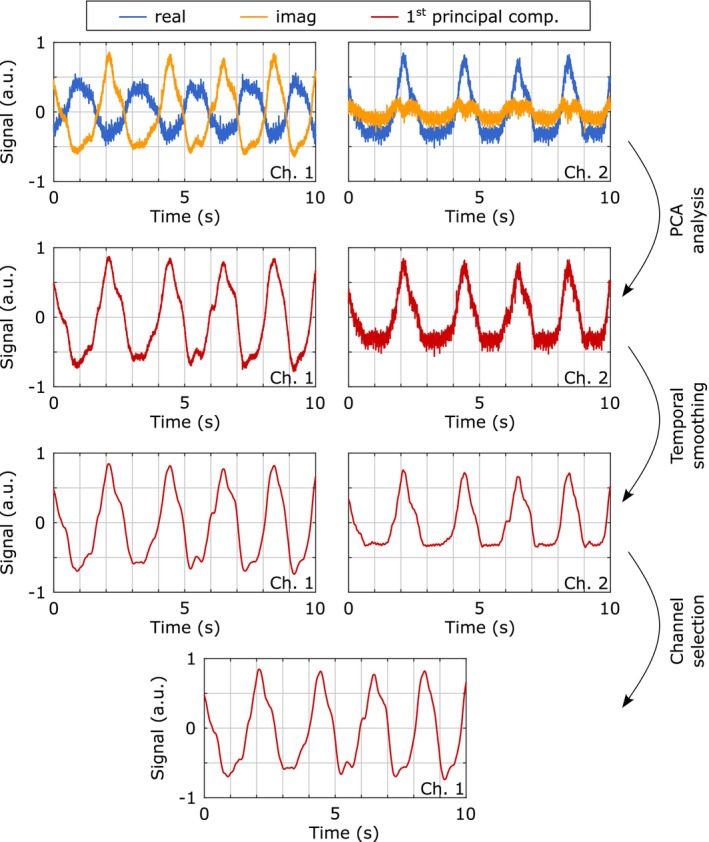
Self‐gating signal processing for respiratory motion binning. The workflow begins with channel‐wise real and imaginary components of averaged self‐gating ADC points. Each channel's data undergoes PCA projection onto its first principal component, followed by temporal smoothing. A quality metric, defined as the ratio of signal power in the 0.1–1.0 Hz band (respiratory motion) to that in the 1.0–10 Hz band (higher frequency noise), determines the selection of the optimal channel as a representative self‐gating signal. For illustrative purposes, only two representative channels are shown.

Respiratory motion binning (Step 2 above) into 24 bins was implemented as follows (cf. Figure 3):

1. Averaging of the complex self‐gating ADC points for each receive channel.
2. Principal component analysis (PCA) to project each channel's self‐gating data onto its first principal component.
3. Temporal smoothing of the principal component signals using a Gaussian filter (0.2 s window for ^1^H, 0.7 s window for ^23^Na) and subsequent subtraction of the data smoothed with a moving average filter (10 s window).
4. Calculation of a quality metric Θ for each smoothed signal, defined as the ratio of signal power within the typical respiratory frequency range (0.1 Hz—1.0 Hz) to the power in higher frequency components (1.0 Hz—10 Hz).
5. Selection of the signal exhibiting the highest Θ as the representative self‐gating signal.
6. Binning of the acquired k‐space data based on the selected self‐gating signal.

Following the 24‐bin reconstruction, the data were grouped into final motion states with a varying number of bins (1, 2, 3, 4, 6, 8, 12, 24) to evaluate the tradeoff between finer binning (potentially reducing motion blurring) and reduced data per motion state (potentially increasing image noise). This grouping was performed using a sliding window approach. For example, for motion states comprising 6 bins, 19 overlapping windows were defined.

To quantify the impact of the respiratory motion binning on the reconstructed images, a sigmoidal line profile was fitted to the dark/light transitions at the boundaries of the simplified kidney model and the surrounding deionized water along the *z*‐direction [33]. Prior to fitting, the signal was averaged along the *x*‐ and *y*‐axes within a cylindrical volume encompassing the kidney model. The resulting 1D signal curve was then fitted with the following logistic function:

$$S\left(z\right)=\frac{a}{1+\mathrm{Exp}\left[\frac{-z+z_{o}}{b}\right]}+c$$

where a represents a scaling factor, c and $z_{0}$ denote offsets, and b is related to the sharpness of the transition, thus serving as a measure of relative image resolution.

### 2.4 In Vivo

Three healthy volunteers were recruited and underwent imaging using the dual‐nuclei coil array with a 30‐min‐long 3D FLASH sequence with a center‐out radial koosh‐ball trajectory with a 2D golden means pattern [30] during free breathing. Data for both ^1^H and ^23^Na nuclei were acquired successively. All imaging was conducted under an FDA Investigational Device Exemption and an institutional review board‐approved protocol. Before imaging, volunteers underwent medical screening and provided signed written consent to participate in the study.

The data reconstruction pipeline followed the steps outlined in the motion phantom study section, with binning of the data into six distinct respiratory motion states using a sliding window approach, where each motion state contained half of the total data. However, the window width of the Gaussian filter used for temporal smoothing of the principal component signals (Step 3 in the respiratory motion binning) of both nuclei was prolonged to 1.5 s for the in vivo data. This adaptation was empirically determined.

## 3 Results

### 3.1 Coil Safety Evaluation

**FIGURE 4:**
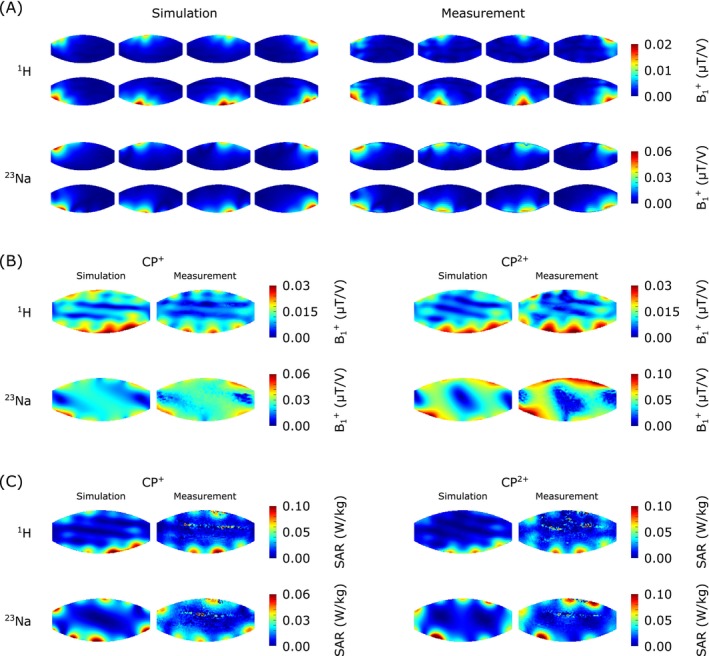
Coil validation results. (A) Simulated and experimentally determined channel‐wise B_1_^+^ magnitude maps for both nuclei. (B) Comparison between simulated and experimental absolute B_1_^+^ maps for both nuclei with CP^+^ and CP^2+^ shimming modes. (C) Local SAR data for both nuclei and shimming modes from simulation and magnetic resonance thermometry experimental studies.

A comparison of the simulated and experimental channel‐wise B_1_^+^ distributions is depicted in Figure 4A for both nuclei. Comparing experimental and simulated B_1_^+^ distributions of the CP^+^ and CP^2+^ modes (Figure 4B), the validation of the coil array's EM model yielded normalized root mean squared error (NRMSE) values of 0.16 and 0.32 for ^23^Na and ^1^H, respectively. Similarly, comparison of the SAR distributions (Figure 4C) resulted in NRMSE values of 0.35 for ^23^Na and 0.22 for ^1^H. The EM simulations of the array positioned on various realistic human body models further provided peak 10 g SAR values when transmitting on the ^1^H frequency of 0.48 W/kg in Duke, 0.41 W/kg in Ella, and 0.43 W/kg in Fats29. Corresponding peak 10 g SAR values for ^23^Na were 0.10 W/kg, 0.16 W/kg, and 0.07 W/kg. Based on these findings, total power limits of 125 W for ^23^Na and 42 W for ^1^H were implemented in the coil file on the scanner console to ensure safe operation in vivo.

### 3.2 Motion Phantom

**FIGURE 5:**
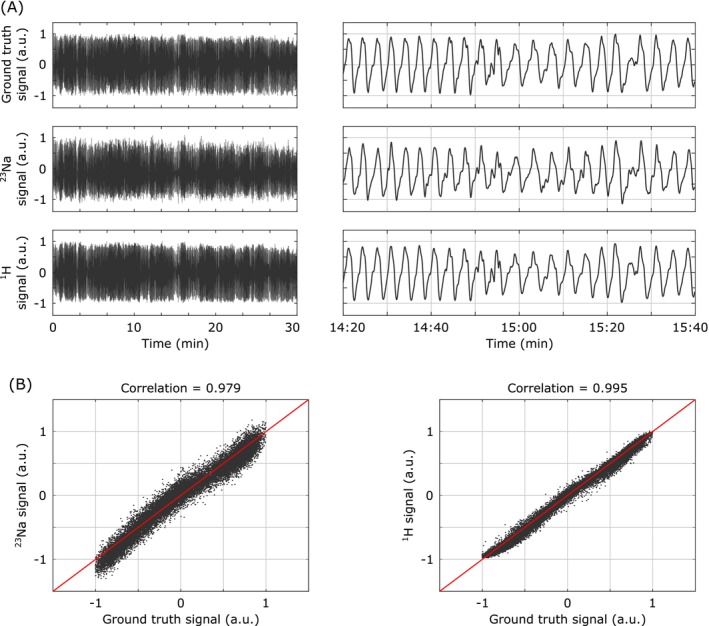
Motion phantom self‐gating validation. (A) Temporal comparison of the ground truth motion input (top) with the acquired ^23^Na (middle) and ^1^H (bottom) self‐gating signals. The left panels show the full 30‐min datasets, while the right panels provide an expanded view of a selected time segment to reveal more detailed signal characteristics. (B) Correlation analysis of the self‐gating signals against the ground truth. The left scatter plot illustrates the relationship for ^23^Na data, and the right for ^1^H data. The red lines represent the ideal identity relationship.

The captured self‐gating signals for both ^23^Na and ^1^H are shown in Figure 5A. Both the ^23^Na (middle row) and ^1^H (bottom row) self‐gating signals closely tracked the ground truth motion (top row) over the full 30‐min acquisition period. Qualitatively, the ^1^H signal showed less noise and matched the ground truth signal in greater detail compared to the ^23^Na signal. This qualitative observation is confirmed in the quantitative correlation analysis depicted in Figure 5B. The ^23^Na self‐gating signal exhibited a correlation coefficient of 0.979 with the ground truth, while the ^1^H signal showed a correlation of 0.995. While both scatter plots confirm the high fidelity of the self‐gating signals in capturing the phantom's motion, the elevated noise in the ^23^Na data leads to a larger spread around the line of identity.

**FIGURE 6:**
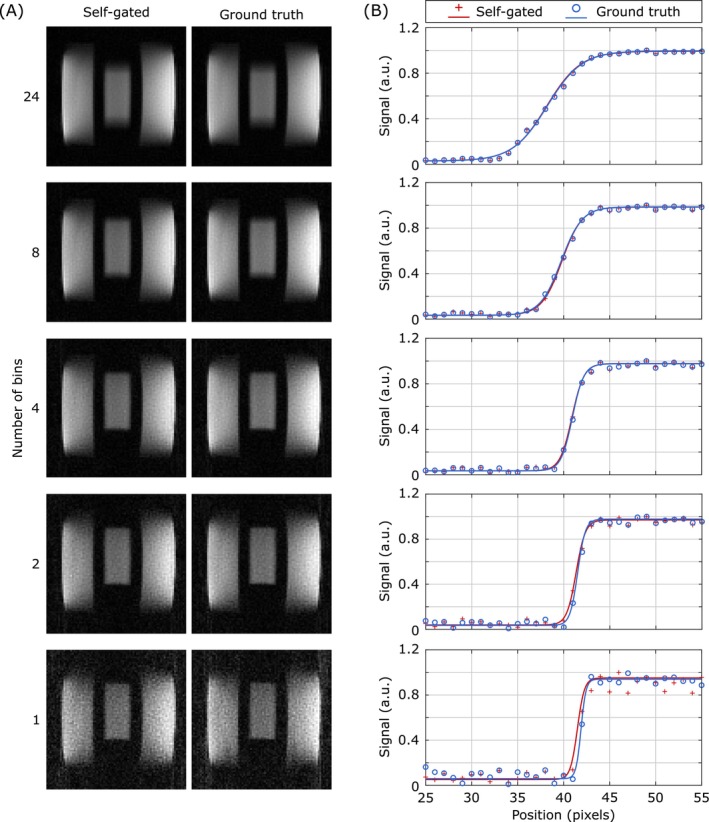
Reconstructed ^23^Na images and resolution assessment from the motion phantom. (A) Representative coronal ^23^Na reconstructions obtained with varying numbers of motion bins (24, 8, 4, 2, 1) consolidated into a single motion state. Images derived from self‐gating‐based binning (left) are compared to those from ground truth‐based binning (right). (B) Quantification of edge sharpness using signal intensity profiles across the kidney model. Measured data points (crosses, circles) and fitted logistic functions (solid lines) demonstrate the impact of motion binning on image quality. The nominal resolution of the images is 3 mm isotropic.

Representative coronal ^23^Na images reconstructed with varying numbers of bins per motion state are presented in Figure 6A. A clear visual improvement in reduced motion blurring was observed as the number of bins per motion state decreased from 24 to 1. Specifically, images reconstructed from a high number of bins (e.g., 24 or 8 bins) exhibited significant blurring. As the number of bins decreased, the edges of the simplified kidney model became notably sharper, indicating better motion compensation. Comparing images derived from self‐gating‐based binning (Figure 6A, left column) with those from ground truth‐based binning (Figure 6A, right column), a high degree of similarity was observed across all binning strategies. Additionally, at the lowest bin count, both self‐gated and ground truth‐binned images showed a significant increase in image noise, consistent with the reduced data per motion state.

**FIGURE 7:**
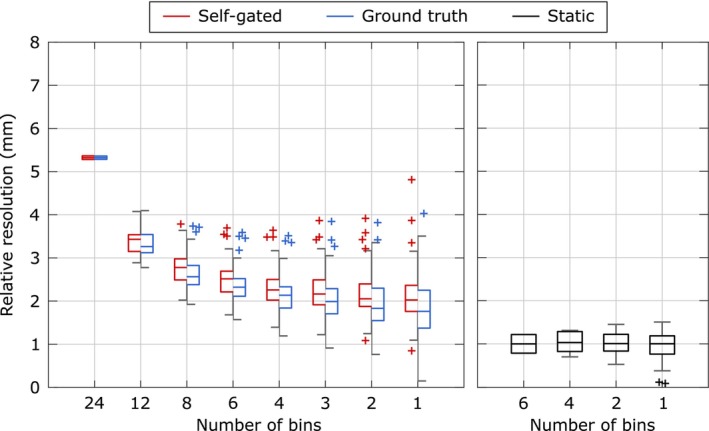
Quantitative assessment of relative image resolution as a function of motion binning strategy. Box plots illustrate the relative image resolution (logistic function parameter *b*) for 24 bins to 1 bin combined into a single motion state. Red boxes represent self‐gating‐based binning, while blue boxes correspond to ground truth‐based binning. For comparison, the right panel shows the relative resolution obtained from a static phantom acquisition (black boxes).

A quantitative assessment of the impact of respiratory motion binning on image quality is shown in Figure 6B with sigmoidal line profiles fitted to the dark/light transitions at the boundaries of the kidney model. Figure 7 summarizes the relative image resolution (logistic function parameter *b*, normalized to the median value of the static acquisitions) as a function of the number of bins per motion state. For both self‐gating‐based (red boxes) and ground truth‐based (blue boxes) binning, a decrease in the number of bins generally led to improved relative resolution values, indicating sharper image transitions. Without motion binning (i.e., combining all 24 bins into a single motion state), the median relative resolution was 5.3. Combining four bins into a single motion state yielded a median relative resolution of 2.3, which was further improved to 2.0 when reconstructing motion states from a single bin based on the self‐gating signal. Importantly, the relative resolution achieved with self‐gating‐based binning closely matched that obtained with ground truth‐based binning. On average, the ground truth‐based binning yielded lower median relative resolutions by 0.2. For comparison, the relative resolution obtained from a static phantom acquisition (black boxes) represents the best‐case scenario without motion. Here, as well as in the motion phantom, an increased number of outliers can be observed with a smaller number of bins per motion state, indicating the impact of increased image noise on the fitting procedure.

### 3.3 In Vivo

**FIGURE 8:**
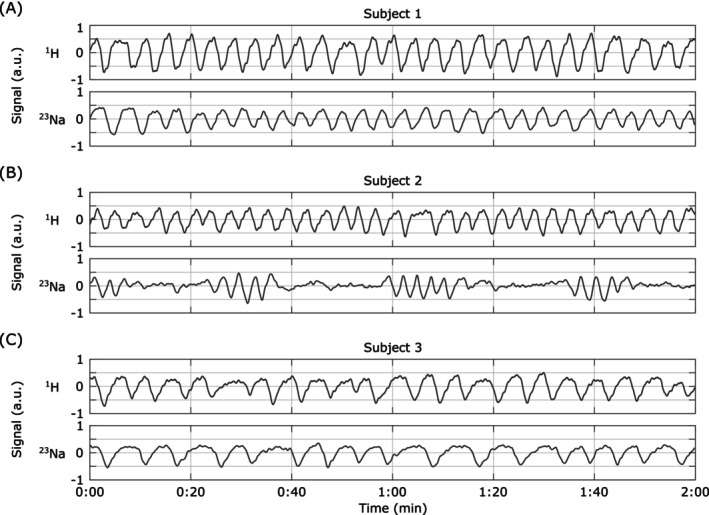
Example in vivo ^1^H and ^23^Na self‐gating signals in three healthy subjects. (A) Subject 1 and (C) Subject 3 exhibit regular breathing patterns. In contrast, (B) Subject 2 fell asleep during the examination and showed severely irregular breathing patterns during the ^23^Na acquisition, likely due to sleep apnea.

Both ^23^Na and ^1^H data were successfully acquired in all three healthy human subjects, demonstrating the self‐gating approach's applicability in vivo. Self‐gating data, indicative of respiratory motion, was extracted for both the ^23^Na and ^1^H acquisitions, spanning the full 30‐min duration for each. Figure 8 presents representative 2‐min segments of this data, clearly illustrating the robust feasibility of capturing respiratory motion in vivo. A noteworthy observation during the acquisition was that Subject 2 fell asleep, leading to a significantly altered respiratory pattern (Figure 8B), characteristic of sleep apnea, which was captured by the self‐gating signal.

**FIGURE 9:**
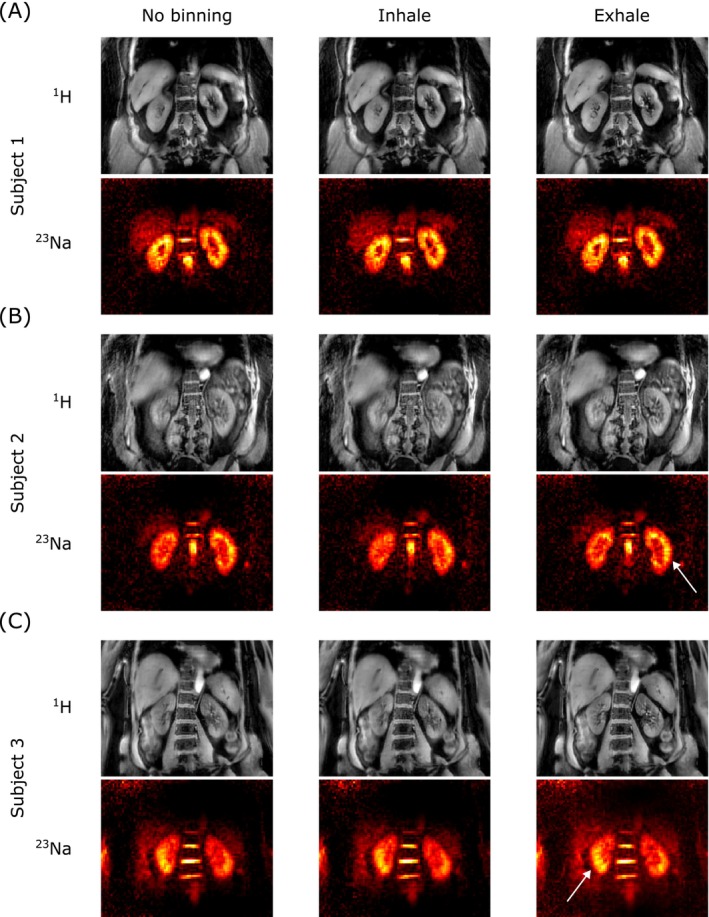
Reconstructed in vivo ^1^H and ^23^Na images from three healthy volunteers. The left column displays images reconstructed without respiratory motion binning. The middle column presents images acquired at end‐inspiration, while the right column shows images acquired at end‐expiration. The white arrows highlight regions with reduced motion‐blurring in the end‐expiration state.

Example coronal slices through the kidneys for all three subjects are presented in Figure 9, providing a visual assessment of the impact of respiratory motion correction. For each subject and nucleus, images are displayed under three conditions: without motion binning (left column), at end‐inspiration (middle column), and end‐expiration (right column). A qualitative assessment of the results shown in Figure 9 revealed that the end‐expiration binned images exhibit sharper anatomical details, compared to the end‐inspiration and the unbinned images. This visual improvement highlights the effectiveness of the self‐gating strategy in compensating for respiratory motion in vivo. Furthermore, animated versions of these results, cycling through all six respiratory motion states, are available in the Supporting Information, offering a more dynamic and comprehensive visualization of the motion compensation.

## 4 Discussion

In this study, we successfully demonstrated the feasibility of in vivo human ^23^Na MRI at 10.5 T, utilizing a novel dual‐tuned ^23^Na‐loop ^1^H‐dipole transceiver array. Our work encompassed the comprehensive design, construction, and electromagnetic characterization of the custom‐built coil array, followed by a detailed evaluation of a self‐gating technique in a controlled motion phantom. Finally, we presented the first in vivo human abdominal ^23^Na MRI results obtained at 10.5 T, highlighting the efficacy of the self‐gating approach in mitigating respiratory motion artifacts.

The development and evaluation of the custom coil array represent a significant aspect of this work. The EM model of the array exhibited good agreement with experimental measurements, comparable to our previously published work on a 16‐channel ^1^H transceiver body array for 10.5 T [18]. This consistent validation across different coil designs at UHF underscores the robustness and reliability of the established EM simulation and experimental characterization methods. While the developed array successfully facilitated dual‐nuclei imaging, its design is primarily optimized for transmission efficiency. The presence of the geometrically decoupled sodium loop resulted in only minor changes in the proton dipole transmit efficiency. While the transmission efficiency with both nuclei was sufficient to perform the described imaging studies, the availability of higher‐rated RF power amplifiers for x‐nuclei and the ability to use independently driven proton channels in a parallel transmit (pTx) platform would benefit sequence optimization and image quality.

To maximize SNR and perform sodium quantification, additional design considerations are needed. Maximizing SNR could be achieved by incorporating a greater number of receive channels beyond the eight transceiver elements implemented in the current study, as routinely implemented for ^1^H imaging at 3 T (118 MHz vs. 123 MHz). This would allow for capturing the full signal available, thereby enabling the full advantage of the high static magnetic field. To accommodate the assessment of sodium concentrations, a strategy is needed to incorporate external reference standards with the close‐fitting array, maintaining performance while appropriately accounting for both receive and transmit fields in the standards and tissue.

The motion phantom study provided crucial validation for the self‐gating approach proposed by Platt et al. [25] in a controlled environment. The strong correlation between the self‐gating signals (for both ^23^Na and ^1^H) and the ground truth motion trajectory confirmed the method's accuracy in capturing respiratory dynamics. This controlled validation was instrumental in fine‐tuning the reconstruction parameters, particularly the respiratory motion binning strategy, which directly informed our approach for the subsequent in vivo human studies. The visual and quantitative improvements in image sharpness, observed in the phantom data, clearly demonstrated the efficacy of this motion compensation strategy. However, the results also indicate that the benefit of binning is somewhat limited. There are trade‐offs that must be balanced between increasing the number of motion states to improve spatial resolution versus decrease in the SNR.

Building on the successful implementation of multi‐nuclear body imaging techniques at 7 T [34, 35, 36, 37, 38], the acquisition of in vivo human ^23^Na and ^1^H data in healthy volunteers at 10.5 T represents a critical step toward multi‐nuclei body imaging at fields above 10 T. The robust self‐gating signals extracted from both nuclei over extended acquisition periods confirm the method's practical applicability. The observation of an altered respiratory pattern in one subject, captured by the self‐gating signal, further underscores the sensitivity and utility of this approach in monitoring physiological motion during prolonged scans. In line with results from Platt et al. [25], qualitative assessment of the motion‐corrected in vivo images revealed noticeably sharper anatomical details, particularly at end‐expiration, providing compelling visual evidence of the self‐gating strategy's effectiveness.

While the self‐gating technique proved feasible for both nuclei, an interleaved acquisition strategy, utilizing the higher SNR of the ^1^H signal for self‐gating the ^23^Na data, would offer significant benefits. The additional ADC samples acquired at the k‐space center for self‐gating inherently prolong TE. This extended TE leads to increased T_2_* signal decay, consequently reducing the already limited SNR of ^23^Na. By leveraging the superior SNR of ^1^H for self‐gating, we could potentially minimize the TE for ^23^Na acquisitions, thereby preserving valuable signal and improving the overall quality of the ^23^Na images. Although we were unable to implement an interleaved acquisition strategy in this study due to limitations of our system's architecture, this approach has been successfully demonstrated by other research groups at 7 T [37, 39, 40, 41]. There is no fundamental reason why such a strategy should not be applicable and beneficial at higher field strengths, such as 10.5 T, and its implementation would represent a valuable advancement for future ^23^Na body MRI studies.

Furthermore, integration of ^23^Na MRI with high‐field ^1^H kidney imaging, as is possible with the current coil, significantly increases the information content of a single study. Several reviews on UHF kidney imaging [42, 43, 44] have highlighted the wide range of benefits associated with increased field strength for ^1^H studies. Techniques such as BOLD imaging, non‐contrast vessel imaging [45], and arterial spin‐labeled perfusion imaging [46] particularly benefit from the enhanced signal, greater susceptibility contrast, and increased T_1_ relaxation times at UHF [47]. This integration requires leveraging advanced parallel transmit functionality, which was not yet available in combination with sodium (or more generally, x‐nuclei) imaging at our 10.5 T system. In separate studies using dedicated ^1^H transceiver coils and parallel transmit functionality, we achieved excellent results at 10.5 T in the human torso [18, 48, 49]. When combined with high‐sensitivity sodium imaging, these advances offer a wealth of potential imaging biomarkers for the study and monitoring of renal diseases and potentially therapeutic interventions.

## 5 Conclusion

This study successfully demonstrated the first in vivo human ^23^Na MRI at 10.5 T, leveraging a novel dual‐tuned transceiver coil array and an effective self‐gating approach for respiratory motion compensation. We validated the coil array's performance through rigorous electromagnetic simulations and experimental measurements, confirming our established methodologies. The motion phantom study successfully proved the feasibility of the self‐gating technique in a controlled environment, directly informing its application in vivo.

## Funding

This work was supported by the National Institute of Biomedical Imaging and Bioengineering (P41 EB027061, R01 EB029985).

## Supporting information


**Figure S1:** Animated in vivo ^1^H (top) and ^23^Na (bottom) images from three healthy volunteers. Following the 24‐bin reconstruction, the data were grouped into six motion states using a sliding window approach, with each motion state comprising 50% of the acquired data. The two extreme motion states (inhale and exhale) correspond to the static images illustrated in Figure 9.

## Data Availability

The data that support the findings of this study are available on request from the corresponding author. The data are not publicly available due to privacy or ethical restrictions.
