# Supplementary figures and images for: First In Vivo 23Na Human Imaging at 10.5 T Using a Combined Sodium‐Proton Transceiver Body Array
Source: Magn Reson Med. 2025 Dec 2;95(4):2429–41. doi: 10.1002/mrm.70201 (PMC12850562; doi:10.1002/mrm.70201)

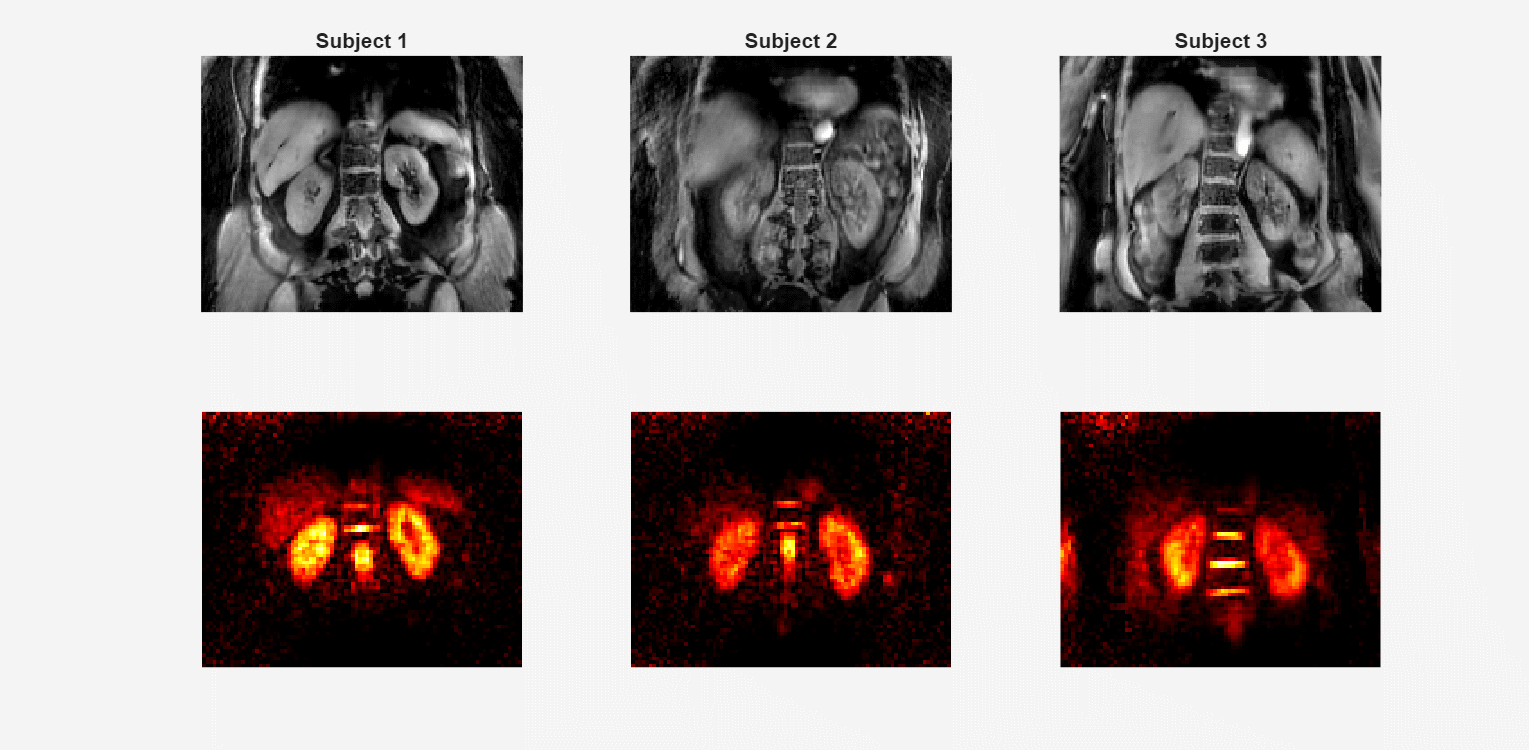

Supplement: Supplementary file 1 — Figure S1: Animated in vivo 1H (top) and 23Na (bottom) images from three healthy volunteers. Following the 24‐bin reconstruction, the data were grouped into six motion states using a sliding window approach, with each motion state comprising 50% of the acquired data. The two extreme motion states (inhale and exhale) correspond to the static images illustrated in Figure 9. [file MRM-95-2429-s001.gif]
